# Long non-coding RNA LINC00520 promotes the proliferation and metastasis of malignant melanoma by inducing the miR-125b-5p/EIF5A2 axis

**DOI:** 10.1186/s13046-020-01599-7

**Published:** 2020-05-28

**Authors:** Wenkang Luan, Yuting Ding, Haitao Yuan, Shaojun Ma, Hongru Ruan, Jinlong Wang, Feng Lu, Xuefeng Bu

**Affiliations:** 1 Department of Plastic Surgery, Affiliated People’s Hospital of Jiangsu University, 8 Dianli Road, Zhenjiang, 212000 Jiangsu China; 2 Department of Rehabilitation, Changshu No. 2 People’s Hospital (The 5th Clinical Medical College of Yangzhou University), Changshu, Jiangsu China; 3 Department of General Surgery, Affiliated People’s Hospital of Jiangsu University, 8 Dianli Road, Zhenjiang, 212000 Jiangsu China

**Keywords:** Melanoma, Growth and metastasis, LICN00520, miR-125b-5p, EIF5A2

## Abstract

**Background:**

Long intergenic non-protein coding RNA 520 (LINC00520), a novel identified lncRNA, has been shown to modulate the malignant phenotype of tumor cells in some malignant tumors. However, the exact role and molecular mechanism of LINC00520 in malignant melanoma has not been studied.

**Methods:**

The expression of LINC00520 in melanoma tissues were detected by using RNA-seq analysis and qRT-PCR. Melanoma cases from the public databases (The Cancer Genome Atlas (TCGA), GEO#GSE15605, GEO#GSE34460 and GEO#GSE24996) were included in this study. CCK-8 assay, EdU assay, transwell and scratch wound assay were used to explore the role of LINC00520 in melanoma cells. Luciferase reporter assays, MS2-RIP, RNA pull-down and RNA-ChIP assay were used to demonstrate the molecular biological mechanism of LINC00520 in melanoma.

**Results:**

We found that LICN00520 was found to be overexpressed in melanoma tissue. High expression of LICN00520 is a risk factor for the prognosis of melanoma patients. LINC00520 promotes the proliferation, invasion and migration of melanoma cells. LICN00520 exerted its oncogenic role by competitive binding miR-125b-5p to promote Eukaryotic initiation factor 5A2 (EIF5A2) expression. We also showed that LICN00520 promotes the growth and metastasis of melanoma in vivo through regulating miR-125b-5p/EIF5A2 axis.

**Conclusions:**

All results elucidated the role and molecular mechanism of LINC00520 in the malignant development of melanoma. LINC00520, a new oncogene in melanoma, maybe serve as a survival biomarkers or therapeutic target for melanoma patients.

## Introduction

Malignant melanoma is the most dangerous skin tumor, which is the primary cause of death of skin cancer [1–3]. In recent years, the global incidence of melanoma is growing rapidly each year [2, 4]. Although melanoma patients are treated by the combination of surgery, chemotherapy, targeted therapy and immunotherapy, the therapeutic effect is still unsatisfactory, especially those with distant metastasis [5]. The development of melanoma is closely related to the abnormal regulation of multiple genes and signaling pathways [6]. Therefore, it is very important to explore the molecular mechanism of the malignant progression of melanoma and to find potential therapeutic targets for melanoma.

Long non-coding RNAs (lncRNAs), a kind of non-coding RNA over 200 nucleotides in length, play pivotal roles in various human tumors by modulating the malignant phenotype of tumor cells [7–9]. A few lncRNAs has been found to be abnormally expressed in melanoma and involved in its malignant progress [10, 11]. Long intergenic non-protein coding RNA 520 (LINC00520), located on chromosome 14, has been reported to overexpress and function as a oncogene in breast cancer, nasopharyngeal carcinoma and laryngeal squamous cell carcinoma [12–14]. It has also been showed that LINC00520 inhibits the growth and metastasis of cutaneous squamous cell carcinoma [15]. However, the exact role and molecular mechanism of LINC00520 in malignant melanoma has not been studied.

In the present study, we found that LINC00520 was highly expressed in melanoma by analyzing the lncRNAs expression profile of melanoma. LINC00520 promoted the proliferation and metastasis of melanoma. To date, the most widely studied mechanism of lncRNAs in tumor is that lncRNAs play the role of competitive endogenous RNAs (ceRNA) in tumor development [16, 17]. Similarly, LINC00520 has been shown to play the same role in nasopharyngeal carcinoma [14]. Here, we have established the ceRNA regulatory network of LINC00520 based on RNA-seq and miRNA-seq results and bioinformatics predictions. We demonstrated that LINC00520 exerts its oncogene effect in melanoma by regulating Eukaryotic initiation factor 5A2 (EIF5A2). EIF5A2, SUPPLlocated on human chromosome 3q25–27, function as a novel oncogene in many tumors [18, 19]. Extensive studies have demonstrated that EIF5A2 participates in the proliferation, migration, invasion and chemotherapeutic resistance of hepatocellular carcinoma, esophageal cancer, gastric cancer, melanoma, etc. [20–23]. We proved that miR-125b-5p exerts anti-cancer effects in melanoma by targeting EIF5A2. Furthermore, we showed that LICN00520 can remove the inhibition effect of miR-125b-5p on EIF5A2 through decoying miR-125b-5p, thus promoting the expression of EIF5A2. Therefore, LICN00520 can serve as a new special diagnostic indicator and therapeutic target in melanoma patients.

## Materials and methods

### Tissue samples

Forty-one primary malignant melanoma tissues and adjacent normal tissues (ANT) were collected from the melanoma patients in the Affiliated People’s Hospital of Jiangsu University, and informed consent was obtained from all patients.. The pathological diagnosis was made independently by two pathologists. None of the patients had undergone chemotherapy or radiotherapy. The study was approved by the Human Research Ethics Committee of the Affiliated People’s Hospital of Jiangsu University. The public database of melanoma from The Cancer Genome Atlas (TCGA), GEO#GSE15605, GEO#GSE34460 and GEO#GSE24996 were also included in this study.

### RNA-seq, miRNA-seq and ceRNA analysis

Three melanoma tissues and adjacent normal tissues were stored in liquid nitrogen, and TRIzol (Invitrogen, USA) was used to extract RNA. Gminix (Shanghai, China) conducted the RNA-seq and miRNA-seq analysis. The network of LICN00520-miRNA-target gene was constructed by using Cytoscape software (v.3.6.0) based on the RNA-seq and miRNA-seq results. The interaction between LINC00520 and miRNAs was predicted through miRcode. TargetScan, miRDB and miRTarBase were used to find the target genes of miRNAs.

### Cell lines and cell culture

Human malignant melanoma cell lines (A375, A2058, MeWo, CHL-1, SK-MEL-28) were obtained from the American Type Culture Collection (ATCC, USA), and growed in Dulbecco’s modified Eagle’s medium (DMEM; Gibco, USA) with 10% fetal bovine serum (Invitrogen, USA). Human epidermal melanocytes (HEMa-LP) was purchased from Invitrogen (USA), and maintained in medium 254 (Cascade Biologics, USA). These cell lines were incubated in the humidified incubator with the atmosphere of 37 °C containing 5% CO^2^.

### Plasmids, oligonucleotides and transfection

The miR-125b-5p mimic, miR-125b-5p inhibitor and related negative control were obtained by GenePharma (Shanghai, China). The small interfering RNA (siRNA) and short hairpin RNA (shRNA) of LINC00520 were also chemically synthesized by GenePharma (Shanghai, China). The EIF5A2 plasmid was constructed by inserting the full length of EIF5A2 into pcDNA3.1 vector (Invitrogen, USA). The shRNA and the control were inserted into the lentivirus vector (GenePharma, Shanghai, China), and the stably expressing sh-LINC00520 shRNA A375 cells were constructed by infecting cells with the corresponding lentivirus. Lipofectamine 3000 (Invitrogen, USA) was used to transfect the related oligonucleotides into melanoma cells.

### Quantitative RT-PCR

TRIzol reagent was used to extract RNA from cells and tissues according to the specified steps (Invitrogen, USA). Fermentas and microRNA reverse transcription kits (Applied Biosystems, CA) were used to conduct reverse transcription. The amplification reactions were conducted by using the ABI StepOnePlus System (Applied Biosystems, CA) according to the set reaction conditions. The special primer of miR-125b-5p was purchased from RiboBio (Guangzhou, China). GAPDH and U6 was used for normalization respectively. The following primers were used: LINC00520 forward 5′-CCTGCTCCTTCAGGGACATC-3′ and LINC00520 reverse 5′-TCCGCCCCTTGCTCAAATAG-3′; EIF5A2 forward 5′-TTCCAGCACTTACCCTT-3′ and EIF5A2 reverse 5′-TTCCCCTCTATTTTTG-3′; GAPDH forward 5′- GTCAACGGATTTGGTCTGTATT-3′ and GAPDH reverse 5′- AGTCTTCTGGGTGGCAGTGAT-3′. The method of 2^–△△Ct^ was used to calculate the relative expression level.

### Western blot

RIPA buffer (KenGEN, China) was used to extract the protein following the appropriate steps. BCA Protein Assay Kit (Beyotime, China) was used to measure the concentration of extracted protein. Western blotting is carried out as the previous described [17]. Antibodies against EIF4A2 (Abcam, 1:1000, Cambridgeshire, UK), vimentin (Abcam, 1:2000, Cambridgeshire, UK), E-cadherin (Abcam, 1:500, Cambridgeshire, UK), N-cadherin (Abcam, 1:1000, Cambridgeshire, UK) was used to the related protein level. β-actin (1:1000, Abcam, UK) and GAPDH (1:2500, Abcam, UK) were used for normalization.

### Cell proliferation assay

For cell counting kit-8 (CCK-8, Beyotime, Shanghai, China) assay, the transfected melanoma cells (5000 cells) were seeded in a 96-well plate, and the process is carried out as described previously [24]. Microplate reader (Multiscan FC, Thermo Scientific) was used to measure the absorbance at an optical density of 450 nm. For EdU assay, the DNA synthesis of melanoma cells grown was measured by using a EdU imaging kit (life Technologies, USA). The assay were carried out according to the manufacturer’s instructions. Immunostaining were visualized by using Leica DMI3000B microscope, and the positive cells were counted.

### Cell invasion and migration assays

Transwell assay was used to detect the invasiveness of melanoma cell. Transfected melanoma cells were digested and resuspended in serum-free DMEM, and were placed at the top of the Matrigel-coated chambers (BD Biosciences, USA). The culture medium with 10% fetal bovine serum was used as the chemical attractant and added to the lower chamber. After 24 h, the fixed invasive cells were stained with crystal violet, counted and photographed. Scratch wound assay was used to evaluate the migration of melanoma cells. Transfected melanoma cells were added into the 6-well plates, and the wound space was formed by the tip of a 200 μl pipette. The width of wound was recorded at 0 and 24 h respectively.

### Isolation of RISC-associated RNA

We used 1% formaldehyde to fix miR-125b-5p overexpressed melanoma cells. We did the chromatin fragmentation. NETN buffer was used to dissolve the cells, the cells were then cultured with Dynabeads protein A (Invitrogen, USA) plus IgG or anti-Pan-Ago, clone 2A8 antibody (Millipore, USA). We used proteinase K digestion to release immunoprecipitated RNA. The extracted RNA was purified by glycogen ethanol precipitation and treated with DNase I.

### Luciferase reporter assay

The fragment of EIF4A2 3′-UTR and LINC00520 containing the miR-125b-5p binding site were inserted into pMIR-REPORT plasmid, and the mutated plasmid the used as the control. The corresponding oligonucleotides and luciferase reporter plasmids were co-transfected into melanoma cells. The luciferase activity of luciferase reporter plasmids was measured by Dual Luciferase Reporter Assay System (Promega, USA) .

### Fluorescence in situ hybridization (FISH)

RiboTM Fluorescent In Situ Hybridization Kit (RiboBio, Guangzhou, China) was used to for FISH. The procedure was carried out according to the previous study [25]. The probe of LINC00520 was synthesized by RiboBio (Guangzhou, China). and the cell nucleus were stained with DAPI. Representative images were obtained by using a confocal microscopy, and the image J software was used to collect signals.

### MS2-RIP assay

Maltose-binding protein (MBP)-affinity purification was used to detect miRNAs that binding to LINC00520. According to the Steitz laboratory steps, MS2-MBP was purified from E. colicoli. 3 bacteriophage MS2 coat protein binding sites were inserted in the downstream of LINC00520 by using Stratagene Quik Change Site Directed Mutagenesis Kit. The MS2-tagged LINC00520 was transfected into the melanoma cell to obtain miRNAs that associated with LINC00520. The RIP analysis was performed on the cells as previously described after 48 h [17], and the miR-125b-5p level was detected by qRT-PCR.

### RNA pull-down assay

The Biotinylated of miR-125b-5p was chemical synthesized by GenePharma (Shanghai, China), and the biotinylated mutant and NC were used as control. The related oligonucleotides were transfected into melanoma cells. The lysates of cells were cultivated with M-280 streptavidin magnetic beads (Invitrogen, USA) [26]. QRT-PCR was used to detect the LINC00520 level in the bound RNA.

### Xenograft tumor and in vivo lung metastasis assay

10 nude mice were obtained from the Beijing Laboratory Animal Center (Beijing, China), and these mice were subcutaneously injected with A375 cells stably expressing LINC00520 siRNA. The volume of tumour was measured every 4 days according to the formula (0.5 × length × width^2^). After 28 days, mice were sacrificed, and tumour tissues were stripped and weighed. We injected A375 cells stably expressing sh-LINC00520 into the tail vein of mice. 10ul/g sterile D-Luciferin firefly potassium salt (Beyotime, China) were intraperitoneal injected into 8 nude mice, and the PerkinElmer IVIS Spectrum (Xenogen, CA) was used for in vivo imaging. The results were quantified by using the Living Image software (Xenogen, CA). After 20 days, the lung was dissected and the metastatic nodules were counted. The study was approved by the Experimental Animal Ethics Committee of the Affiliated People’s Hospital of Jiangsu University.

### Immunohistochemistry staining and HE staining

Immunohistochemistry is performed as described previously [27] using the antibody EIF4A2. The optical density of the image was analyzed by image J software. For HE staining, the sections were deparaffinizated and rehydrated. Then, the sections were incubated with hematoxylin and stained in acid ethanol and eosin. The sections were dehydrated with alcohol and cleared with xylene. Representative images were taken with a microscope.

### Statistical analysis

Data expressed as mean ± SD. SPSS13.0 was used to analyse the data. Data was evaluated by t-test or one-way ANOVA, and spearman correlation analysis was analysed by using the MATLAB. Kaplan-Meier survival curves was used to evaluate the relationship between LINC00520 expression and melanoma patient survival. Melanoma tissues were separated into two groups according to the expression of LINC00520, the differences between the curves were tested by the log-rank test. GraphPad Prism was used to plot the Kaplan-Meier survival curves. *P* value < 0.05 is statistically significant.

## Results

### LINC00520 was significantly up-regulated in melanoma

**Fig. 1:**
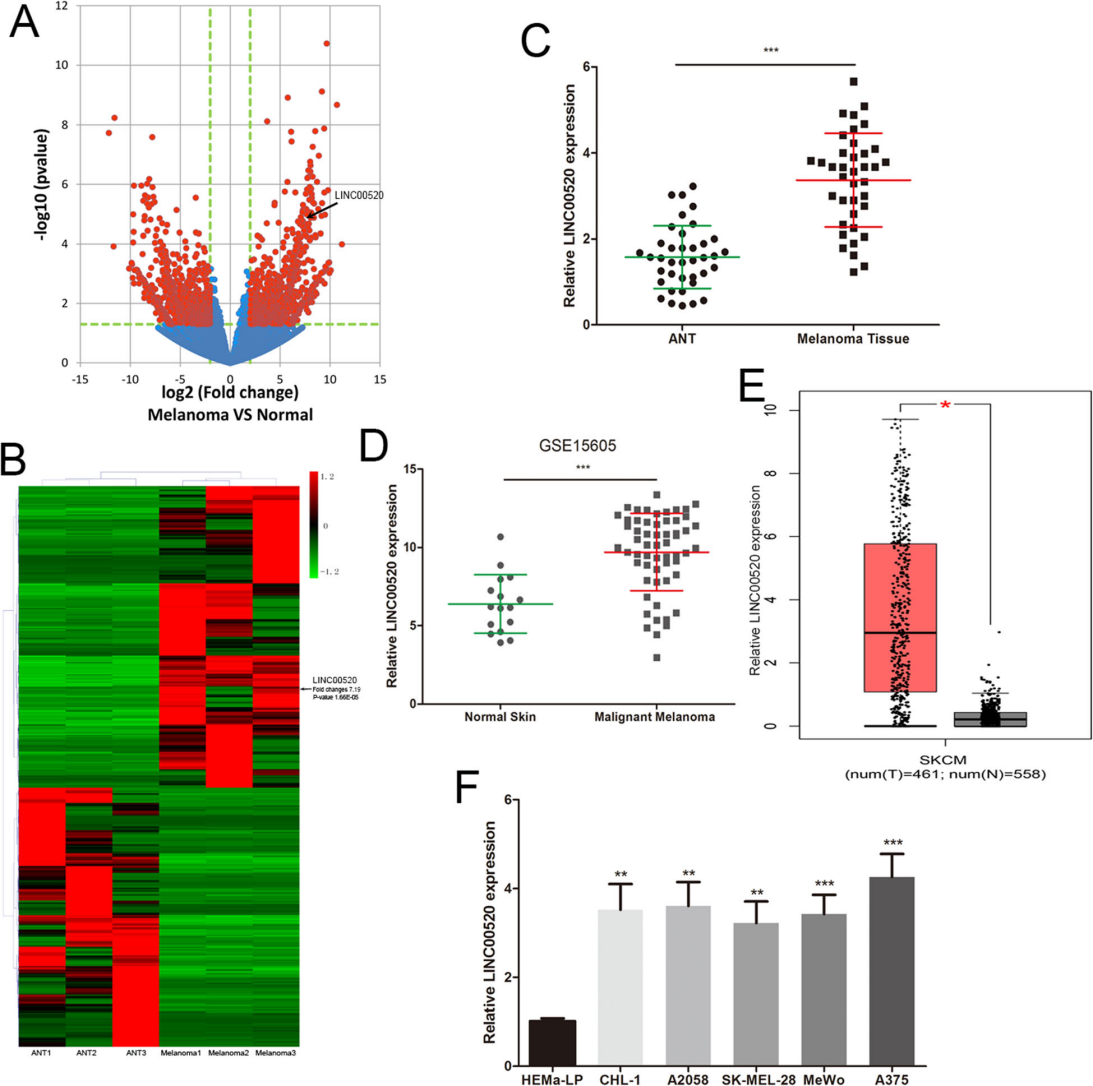
LINC00520 was significantly up-regulated in melanoma. **a** The volcano plot showed the levels of lncRNAs between primary malignant melanoma tissues and ANT. The vertical lines represent 2.0-fold changes, and the horizontal line represents *P*-value of 0.05. The red dot correspond to the differentially expressed lncRNAs with statistical significance. **b** The cluster heat map showed differentially expressed lncRNAs over 3.0-fold change in melanoma tissues. **c** The level of LINC00520 was analyzed in 38 malignant melanoma tissues and ANT. **d** The LINC00520 levels were detected in the GEO#GSE15605 dataset. **e** GEPIA (http://gepia.cancer-pku.cn/) was used to detect the expression of LINC00520 in TCGA melanoma dataset. **f** The expression profile of LINC00520 in human melanoma cell lines (A375, A2058, MeWo, CHL-1, SK-MEL-28) and human epidermal melanocytes (HEMa-LP). Data were expressed as the mean ± SD, *P < 0.05, **P < 0.01, ***P < 0.001

We first analysed the lncRNA expression profiling in three malignant melanoma tissues and three adjacent normal tissues (ANT) by using RNA-seq. Volcano plots showed the differentially expressed lncRNAs over 2.0-fold change between melanoma tissues and ANT (Fig. 1a). All lncRNAs whose expression changes over 3.0-fold were shown in a cluster heat map (Fig. 1b). Thereinto, the LINC00520 level was up-regulated 7.19-fold in melanoma tissues (Fig. 1b). We verified the RNA-seq results by detecting the expression of LINC00520 in 38 melanoma tissues and ANT, and found that LINC00520 was increased in melanoma tissues (Fig. 1c). We also found the same result by analyzing the published datasets (GEO#GSE15605) (Fig. 1d). GEPIA (http://gepia.cancer-pku.cn/) was used to analyze the expression of LINC00520 in melanoma dataset of The Cancer Genome Atlas (TCGA), and found that LINC00520 was overexpressed in melanoma (Fig. 1e). Meanwhile, the level of LINC00520 in malignant melanoma cell (A375, A2058, CHL-1, MeWo, SK-MEL-28) was higher than that in human epidermal melanocytes (HEMa-LP) (Fig. 1f). These suggested that LINC00520 maybe participate in the malignant development of melanoma.

### LINC00520 is an risk factor for the survival of patients with melanoma

**Fig. 2:**
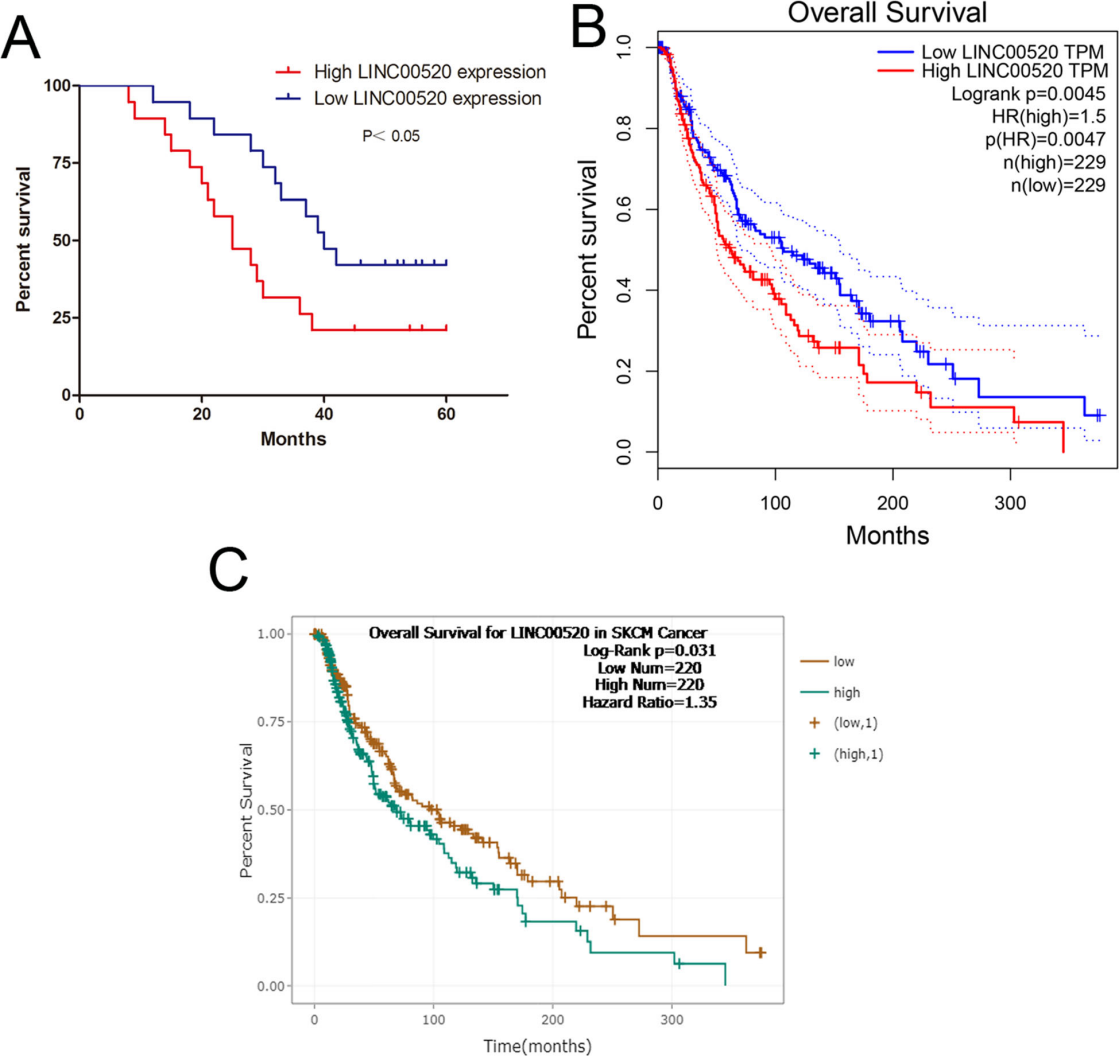
LINC00520 is an risk factor for the survival of patients with melanoma. **a** The overall survival curves of 38 melanoma patients with high and low LINC00520 levels. **b** The prognostic data of melanoma in TCGA was analyzed by using GEPIA (http://gepia.cancer-pku.cn/). **c** We analyzed the prognostic data of melanoma in TCGA by using Starbase (http://www.sysu.edu.cn). Data were expressed as the mean ± SD

We investigated the clinical significance of LINC00520 in melanoma patients. The high expression of LINC00520 (expression ratio ≥ median ratio) is closely related to the clinical stage of melanoma, but not to age, sex, ulcer and family history (Table 1). In our melanoma patient samples, Kaplan-Meier analysis showed that the survival rate of melanoma patients with high LINC00520 levels was poorer (Fig. 2a). We next analyzed the melanoma patients prognostic data of TCGA by using GEPIA (http://gepia.cancer-pku.cn/) and Starbase (http://www.sysu.edu.cn), and found that the high LINC00520 levels were correlated with poor survival rate of melanoma patients (Fig. 2b and c). These demonstrated that high LINC00520 expression is an risk factor for the melanoma patients.

**Table 1:** Correlation between LINC00520 levels and clinical pathological characteristic (*n* = 38)

Clinical characteristics	Number	High LINC00518 expression	Low LINC00518 expression	*P*-value
**Age**				0.494
<50	13	5	8	
≥ 50	25	14	11	
**Gender**				0.742
Male	22	12	10	
Female	16	7	9	
**Family history**				0.403
Yes	7	2	5	
No	31	17	14	
**Ulcer**				0.330
Yes	20	12	8	
No	18	7	11	
**TMN stage**				<0.01
I-II	14	2	12	
III	24	17	7

### LINC00520 promotes the proliferation, invasion and migration of melanoma cell

**Fig. 3:**
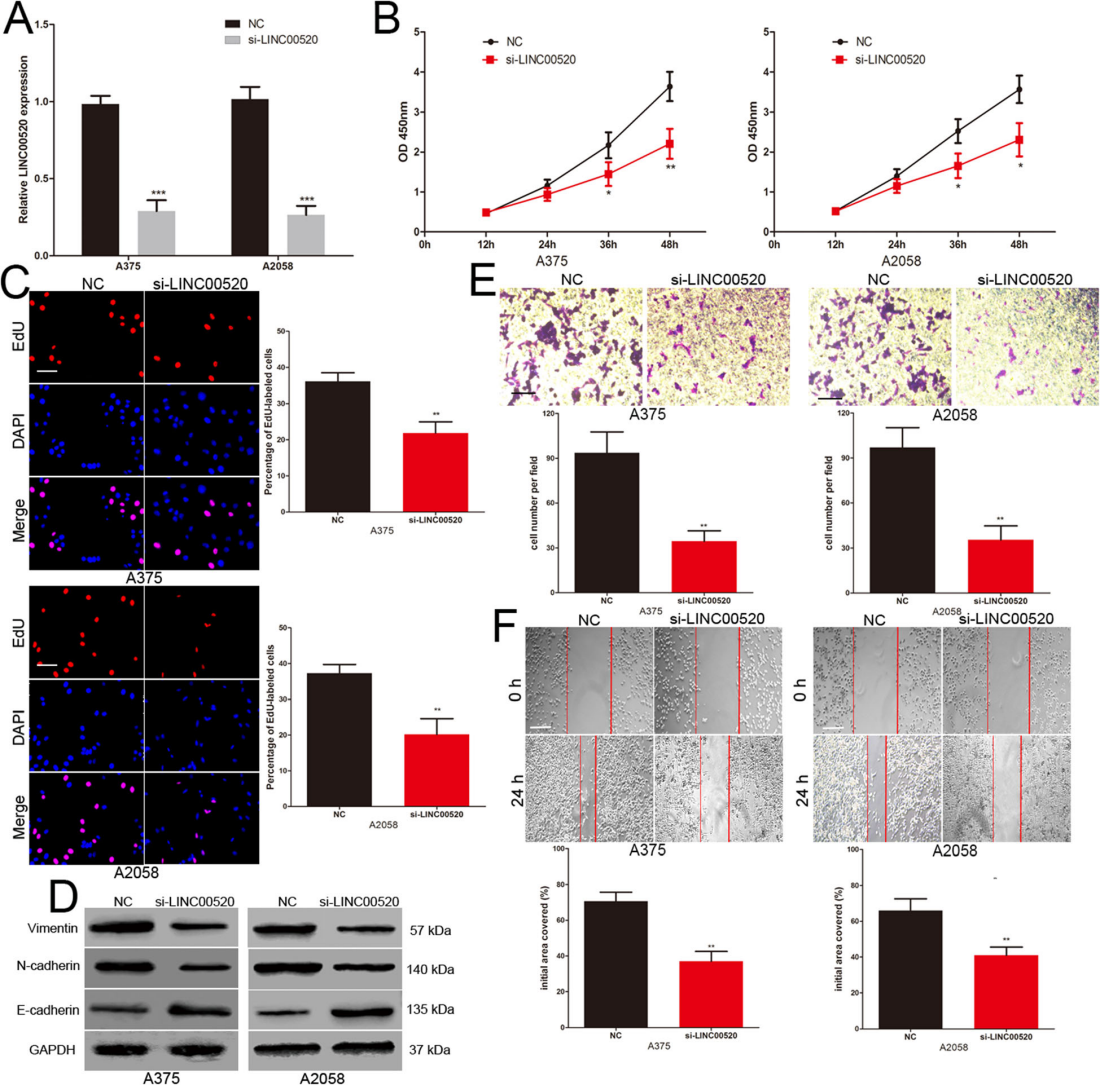
LINC00520 promotes the proliferation, invasion and migration of melanoma cell. **a** Transfection efficiency of LINC00520 siRNA was determined by PCR. **b** The proliferative ability of melanoma cells was determined by CCK8 assay in different groups. **c** The DNA synthesis of melanoma cells grown was detected by EdU assay after transfection with NC or LINC00520 siRNA. Scale bar, 100 μm. **d** Western blots identified N-cadherin, E-cadherin and Vimentin protein expression changes in NC or LINC00520 siRNA transfected melanoma cells, GAPDH was used as a control. **e** The invasive capacity of NC or LINC00520 siRNA transfected melanoma cells was assessed by transwell assay. Scale bar, 50 μm. **f** Migration of melanoma cells in different transfection groups was detected by scratch wound assay. Scale bar, 100 μm. Data were expressed as the mean ± SD, *P < 0.05, **P < 0.01, ***P < 0.001

To explore the influence of LINC00520 on the biological role of melanoma cell, the LINC00520 siRNA was transfected into A375 and A2058 cells (Fig. 3a). CCK-8 assays revealed that reduction of LINC00520 significantly repressed the proliferation ability of A375 and A2058 cells (Fig. 3b). EdU assay showed that the number of EdU-positive cells in LINC00520 knockdown melanoma cells were significantly reduced compared with the control group (Fig. 3c). Increasing evidence show that epithelial-to-mesenchymal transition (EMT) is a key event in the process of tumor metastasis [28]. In EMT, there are morphological changes epithelial-like to mesenchymal-like appearance [29]. We explored the effects of LINC00520 on the EMT of melanoma cells. The level of epithelial cell marker (E-cadherin) was increased, whereas the levels of the mesenchymal markers (N-cadherin and vimentin) were decreased in LINC00520 knockdown melanoma cells (Fig. 3d). Transwell assays demonstrated that LINC00520 siRNA inhibited the invasive capacity of A375 and A2058 cells (Fig. 3e). Scratch wound assays revealed that the migrative capacity of melanoma cells was suppressed by the LINC00520 siRNA (Fig. 3f). In addition, A375 and A2058 are BRAF mutated melanoma cells. We repeated cell proliferation, invasion and migration experiments with BRAF-WT MeWo cells and reached the same conclusion (Supplementary Fig. 1B-D). This suggested that the role of LINC00520 in melanoma cells is independent of BRAF mutation.

### ceRNA analysis of LINC00520

**Fig. 4:**
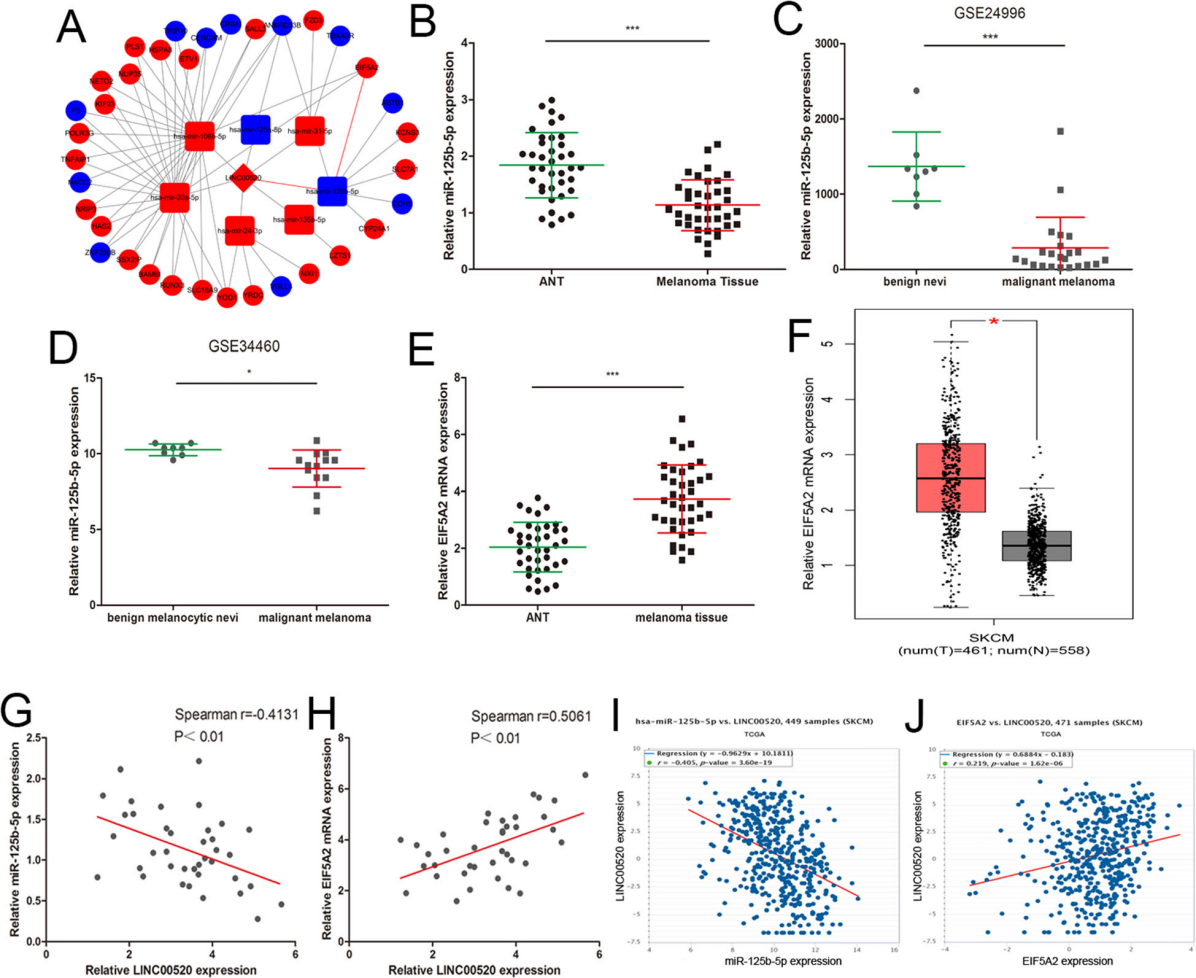
ceRNA analysis of LINC00520. **a** Cytoscape was used to visualize LINC00520-miRNA-target gene nectworks based on our miRNA-seq and RNA-seq data. The interaction between LINC00520 and miRNAs was predicted through miRcode. TargetScan, miRDB and miRTarBase were used to find the target genes of miRNAs. Red color correspond to high expression, and blue color correspond to low expression. **b** The miR-125b-5p expression were detected in 38 malignant melanoma tissues. **c** The expression of miR-125b-5p was analyzed by using GEO#GSE24996 dataset. **d** The expression of miR-125b-5p was detected in GEO#GSE34460 dataset. **e** The expression of EIF5A2 was analyzed in 38 malignant melanoma tissues and ANT. **f** The expression of EIF5A2 was detected from TCGA by using GEPIA (http://gepia.cancer-pku.cn/). **g** The correlation of LINC00520 and miR-125b-5p in 38 melanoma tissues was negative. **h** The positive correlation between LINC00520 and EIF5A2 mRNA in 38 melanoma tissues. **i** A negative correlation between LINC00520 and miR-125b-5p expression were found in TCGA melanoma dataset. **j** The positive correlation between LINC00520 and EIF5A2 mRNA levels in TCGA melanoma dataset. Data were expressed as the mean ± SD, *P < 0.05, ***P < 0.001

We next explored the molecular mechanism of LINC00520 in melanoma. LINC00520 has been shown to play its role in nasopharyngeal carcinoma through the ceRNA mechanism [14]. We then constructed the LINC00520-miRNA-target gene network based on our miRNA-seq and RNA-seq data (Fig. 4a). The interaction between LINC00520 and miRNAs was identified using miRcode, and the target genes of the miRNAs were predicted through TargetScan, miRDB and miRTarBase. Cytoscape was used to visualize the interrelationships of LINC00520-miRNA-target gene. In the network, the miR-125b-5p/EIF5A2 axis caught our attention because of miR-125b-5p was decreased and EIF5A2 was increased in melanoma tissue (Fig. 4a). In order to further verify the results of ceRNA analysis, the expression of miR-125b-5p and EIF5A2 were detected in 38 melanoma tissues and ANT. The level of miR-125b-5p was down-regulated in melanoma tissues (Fig. 4b), and the same result were obtained by analysing the previously published dataset (GEO#GSE34460 and GEO#GSE24996) (Fig. 4cand d). EIF5A2 was increased in melanoma tissues compared to ANT (Fig. 4e), and the same result were discovered by analyzing the TCGA-melanoma dataset (Fig. 4f). In our 38 melanoma tissue samples, LINC00520 and miR-125b-5p levels were inversely correlated, while LINC00520 and EIF5A2 levels were positively correlated (Fig. 4g and h). Moreover, the TCGA-melanoma dataset also reveals the same correlation analysis results (Fig. 4i and j).

### LINC00520 sponges miR-125b-5p in melanoma cells

**Fig. 5:**
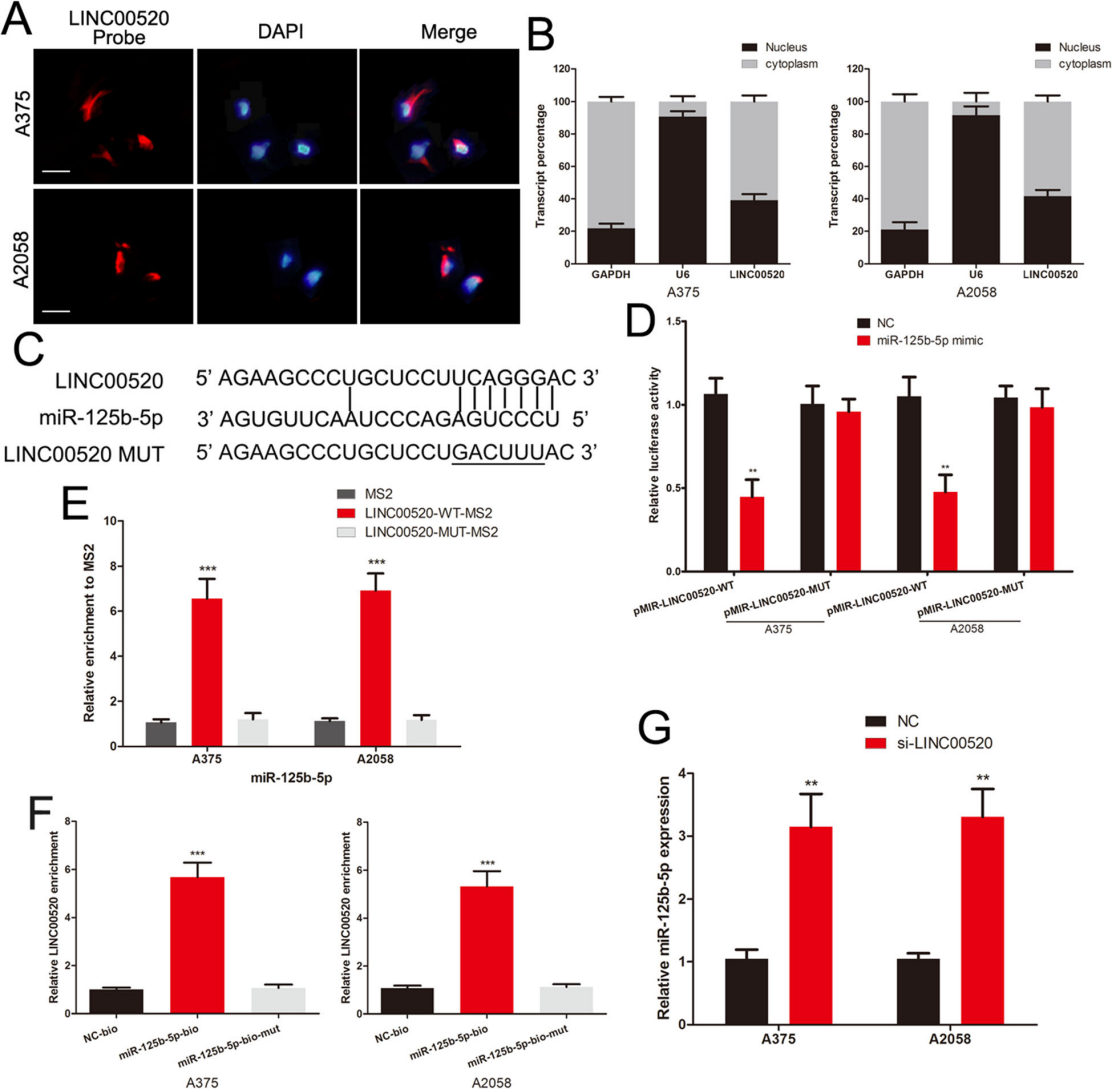
LINC00520 sponges miR-125b-5p in melanoma cells. **a** FISH showed that LINC00520 was mainly distributed in both cytoplasm and nucleus in melanoma cells. Scale bar, 25 μm. **b** qRT-PCR of nuclear and cytoplasm RNA fractions detected the LINC00520 expression in cytoplasm and nuclear. **c** The binding sites of miR-125b-5p on LINC00520, and target sequences were mutated. **d** Luciferase assay of melanoma cells transfected with LINC00520-WT or LINC00520-MUT reporter together with miR-125b-5p or NC. **e** MS2-RIP was used to detect the endogenous miR-125b-5p associated with the MS2-tagged LINC00520. **f** Melanoma cells transfected with biotin-labeled miR-125b-5p, mutated or NC oligos, and assayed by biotin-based pull down. The expression of LINC00520 were detected by qRT–PCR. **g** The levels of miR-125b-5p in melanoma cells following transfection with LINC00520 siRNA or NC. Data were expressed as the mean ± SD, **P < 0.01, ***P < 0.001

We further explored whether LINC00520 can directly binds to miR-125b-5p. LINC00520-FISH and qRT-PCR of nucleus and cytoplasm fragments showed that LINC00520 was distributed in both cytoplasm and nucleus in melanoma cells (Fig. 5a and b). We constructed the LINC00520 luciferase reporter plasmids containing the miR-125b-5p binding sites, and the mutated plasmid was used as the control. (Fig. 5c). The luciferase activity of wild-type LINC00520 vector was significantly inhibited by the miR-125b-5p mimic in melanoma cells, but not the mutant plasmid (Fig. 5d). We subsequently verify the direct binding interaction between LINC00520 and miR-125b-5p using MS2-RIP and RNA pull-down assay. The MS2-tagged wild-type LINC00520 vector enriched lots of miR-125b-5p compared with the empty and mutant plasmids (Fig. 5e). Additionally, RNA pull-down assay also revealed that LINC00520 was pulled down by biotin-labelled miR-125b-5p (Fig. 5f). The level of miR-125b-5p was increased in LINC00520 knockdown A375 and A2058 cells (Fig. 5g). All results suggested that LINC00520 directly binds to miR-125b-5p in melanoma.

### LINC00520 acts as a ceRNA to promote EIF5A2 expression

**Fig. 6:**
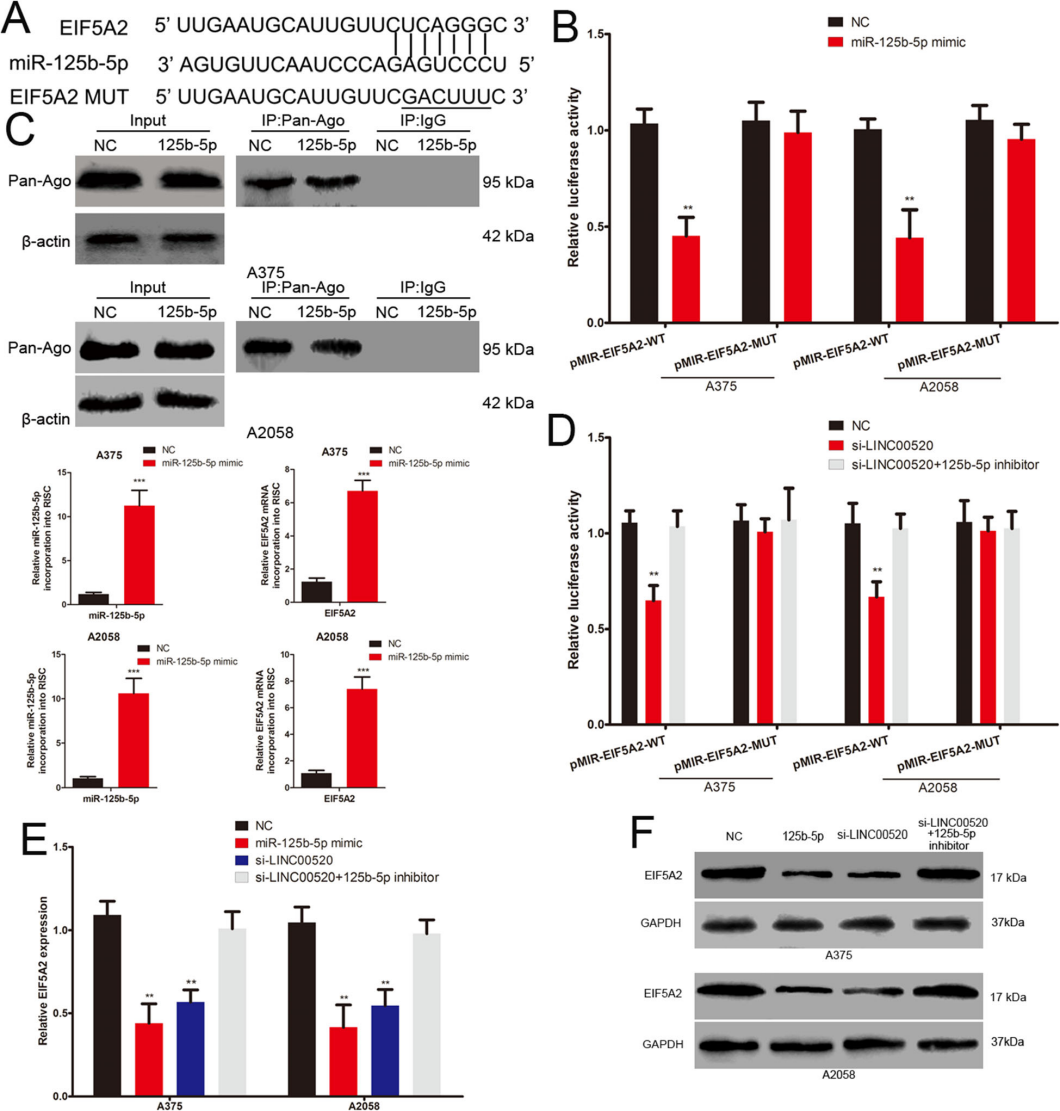
LINC00520 acts as a ceRNA to promote EIF5A2 expression. **a** The binding sites of miR-125b-5p on the 3′-UTR of EIF5A2, and target sequences were mutated. **b** Luciferase assay of cells transfected with EIF5A2–3′ UTR-WT or EIF5A2–3′ UTR-MUT reporter together with miR-125b-5p mimic or NC. **c** Immunoprecipitation (up) of the Ago2/RISC using the Pan-Ago2 antibody in overexpressing miR-125b-5p melanoma cells. IgG was used as a negative control and β-actin was used as an internal control. PCR analysis (down) of miR-125b-5p and EIF5A2 incorporated into RISC in overexpressing miR-125b-5p melanoma cells. **d** Luciferase assay of cells transfected with EIF5A2–3′ UTR-WT or EIF5A2–3′ UTR-MUT reporter together with LINC00520 siRNA or LINC00520 siRNA plus miR-125b-5p inhibitor. **e** The expression of EIF5A2 mRNA in melanoma cells transfected with miR-125b-5p mimic, LINC00520 siRNA or LINC00520 siRNA plus miR-125b-5p inhibitor. **f** Western blots identified EIF5A2 protein expression changes in different groups, GAPDH was used as a control. Data were expressed as the mean ± SD, **P < 0.01, ***P < 0.001

We further discussed the role and mechanism of LINC00520 on EIF5A2 expression. The 3′-UTR of EIF5A2 has the same miR-125b-5p binding sites that miR-125b-5p binds to LINC00520 (Fig. 6a). We constructed the wild-type and mutant EIF5A2 3’UTR luciferase plasmids, and found that the luciferase activity of wild type plasmid was suppressed by miR-125b-5p mimic (Fig. 6b). miRNA transfer into Ago2 protein to form a Ago2/RNA-induced silencing complex (RISC) [30]. miRNAs target the binding of RISC to specific mRNA, leading to mRNA silencing or destabilizing [30]. RNA-ChIP analysis was used to detect the EIF5A2 mRNA abundance in the Ago2/RISC after up-regulation of miR-125b-5p. miR-125b-5p overexpression melanoma cells showed the enrichment of the miR-125b-5p and EIF5A2 level that incorporated into RISC (Fig. 6c). Meanwhile, miR-125b-5p inhibited the expression of EIF5A2 in both mRNA and protein level. (Fig. 6e and f). These demonstrated that EIF5A2 is the target gene of miR-125b-5p. Furthermore, LINC00520 siRNA also led a significantly decrease in the luciferase activity of wild type EIF5A2 3’UTR luciferase plasmid, and this effect could be attenuated by miR-125b-5p inhibitor (Fig. 6d). The mRNA and protein levels of EIF5A2 were repressed by LINC00520 siRNA in melanoma cells, and this inhibition effect could be reversed by miR-125b-5p inhibitor (Fig. 6e and f). Take together, these results demonstrated that LINC00520 promotes EIF5A2 expression by sponging miR-125b-5p in melanoma.

### LINC00520 promotes the growth and metastasis of melanoma cell through miR-125b-5p/EIF5A2 axis

**Fig. 7:**
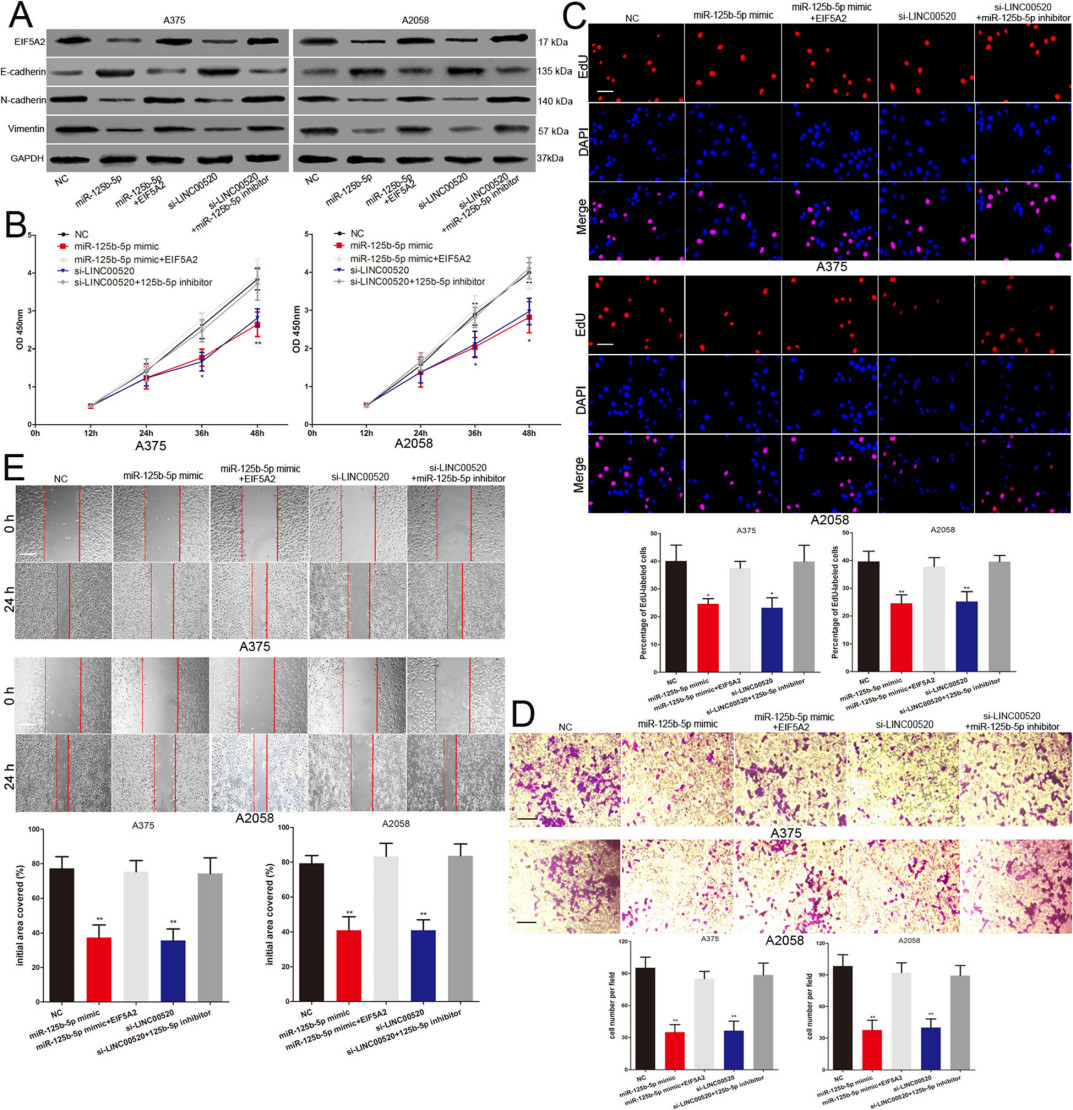
LINC00520 promotes the growth and metastasis of melanoma cell through miR-125b-5p/EIF5A2 axis. **a** Western blots identified EIF5A2, N-cadherin, E-cadherin and Vimentin protein expression changes in NC, miR-125b-5p mimic, miR-125b-5p mimic plus EIF5A2, si-LINC00520 or si-LINC00520 plus miR-125b-5p inhibitor transfected melanoma cells, GAPDH was used as a control. **b** Effect of miR-125b-5p mimic and si-LINC00520 on the proliferative ability of melanoma cells was determined by CCK8 assay. The results were further confirmed by co-transfection EIF5A2 plasmid and miR-125b-5p inhibitor respectively. **c** The DNA synthesis of melanoma cells grown was detected by EdU assay following transfection with NC, miR-125b-5p mimic, miR-125b-5p mimic plus EIF5A2, si-LINC00520 or si-LINC00520 plus miR-125b-5p inhibitor. Scale bar, 100 μm. **d** Effect of miR-125b-5p mimic and si-LINC00520 on the invasive capacity of melanoma cells was assessed by transwell assay. The results were verified by the recovery experiment of co-transfection EIF5A2 plasmid and miR-125b-5p inhibitor respectively. Scale bar, 50 μm. **e** Migration of melanoma cells was detected by scratch wound assay. EIF5A2 plasmid and miR-125b-5p inhibitor reversed the effect of miR-125b-5p mimic and si-LINC00520 on the migration capability of melanoma cells respectively. Scale bar, 100 μm. Data were expressed as the mean ± SD, *P < 0.05, **P < 0.01

EIF5A2 has been shown to act as a new oncogene in many tumors, including melanoma [21–23]. We discussed whether LINC00520 exerts its oncogenic effect in melanoma by regulating EIF5A2 expression. LINC00520 siRNA, miR-125b-5p mimic, miR-125b-5p mimic together with EIF5A2 plasmid and LINC00520 siRNA together with miR-125b-5p inhibitor were transfected into melanoma cells. Western blotting was used to detect the change of EIF5A2 expression in different treatment groups (Fig. 7a). The miR-125b-5p mimics inhibited the proliferation, EMT, invasion and migration of melanoma cells, and this effect were attenuated by the EIF5A2 plasmid (Fig. 7a-e). These suggested that miR-125b-5p plays the role of tumor suppressor in melanoma by targeting EIF5A2. Moreover, we found that the inhibitory effect of LINC00520 siRNA on the EIF5A2 expression, proliferation, EMT, invasion and migration of melanoma cells were reversed by miR-125b-5p inhibitor (Fig. 7a-e). We also found that miR-125b-5p inhibitor abolish the role of LINC00520 siRNA on the EIF5A2 expression, proliferation and metastasis of BRAF-WT melanoma cells (Supplementary Fig. 1A-D). This results further strengthen our conclusions in the context of melanoma independently from the BRAF mutated. Take together, these results indicated that LINC00520 promotes the growth and metastasis of melanoma by decoying miR-125b-5p to promote EIF5A2 expression.

### LINC00520 exerts its pro-growth and pro-metastasis activity through regulating miR-125b-5p/EIF5A2 axis in vivo

**Fig. 8:**
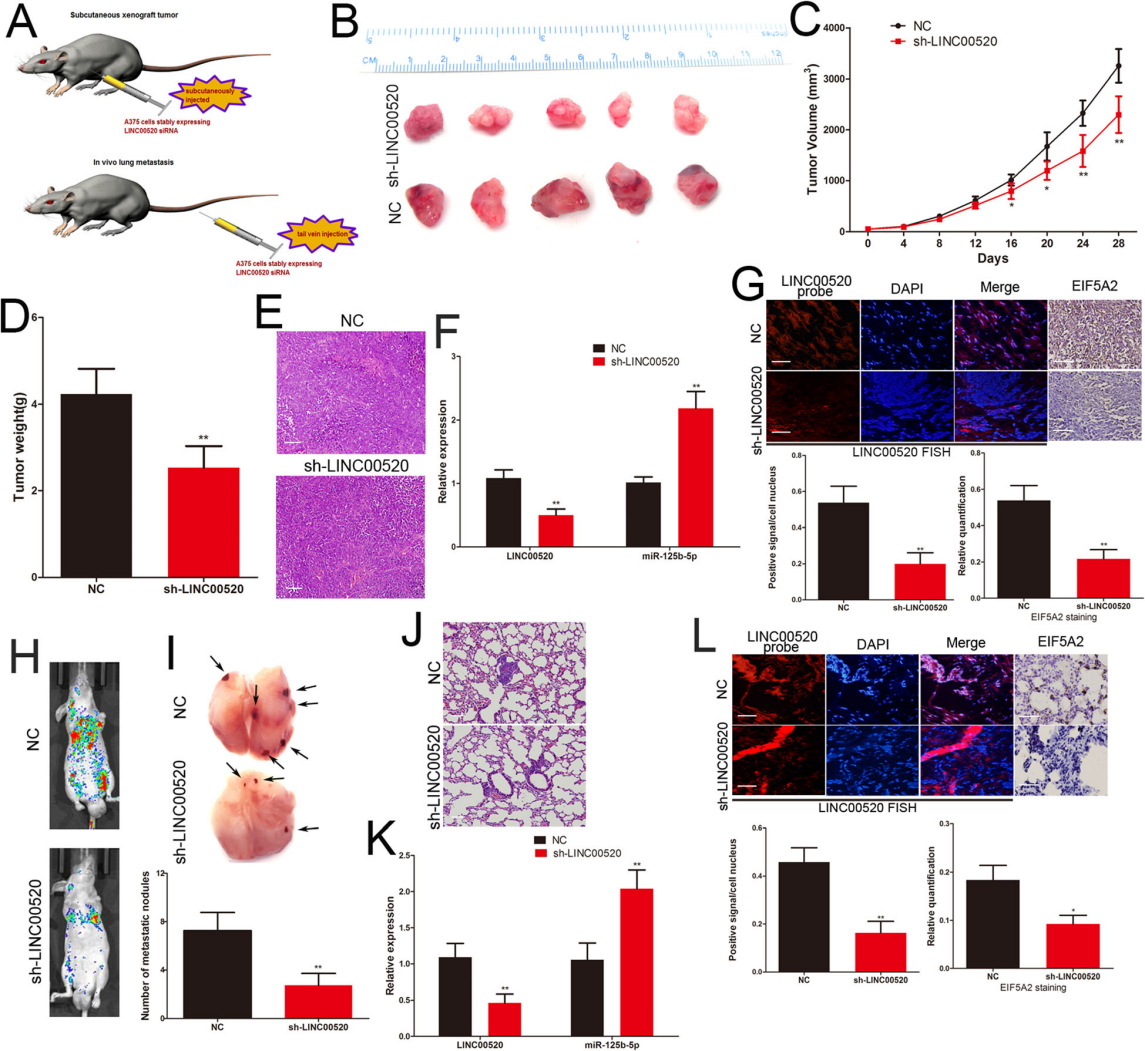
LINC00520 exerts its pro-growth and pro-metastasis activity through regulating miR-125b-5p/EIF5A2 axis in vivo. **a** The schema of the animal experiment. **b** The excision tumor in nude mice of A375 cells that stably expressing LINC00520 shRNA or control xenografts. **c** Differences in the volume of tumor among groups. **d** The tumor weight of excised tumor tissues. **e** Xenograft tumour tissues were confirmed by H&E staining. Scale bar, 50 μm. **f** Expressions of LINC00520 and miR-125b-5p in xenograft tumour tissues were detect by qRT-PCR. **g** The expression of LINC00520 and EIF5A2 were examined by FISH and immunohistochemistry of sections from the xenograft tumour tissues. Scale bar, 25 μm. **h** Representative bioluminescence images of mice after tail vein injection of stably expressing LINC00520 shRNA or control A375 cells. **i** The excision lung tissues in nude mice, LINC00520 shRNA caused a decrease in the number of metastatic lung nodules. **j** Metastatic lung nodules were confirmed by H&E staining. Scale bar, 50 μm. **k** qRT-PCR detected the LINC00520 and miR-125b-5p level in the metastatic lung nodules. **l** The expression of LINC00520 and EIF5A2 were detected by FISH and immunohistochemistry of sections from the metastatic lung nodules. Scale bar, 25 μm. Data were expressed as the mean ± SD, *P < 0.05, **P < 0.01

**Fig. 9:**
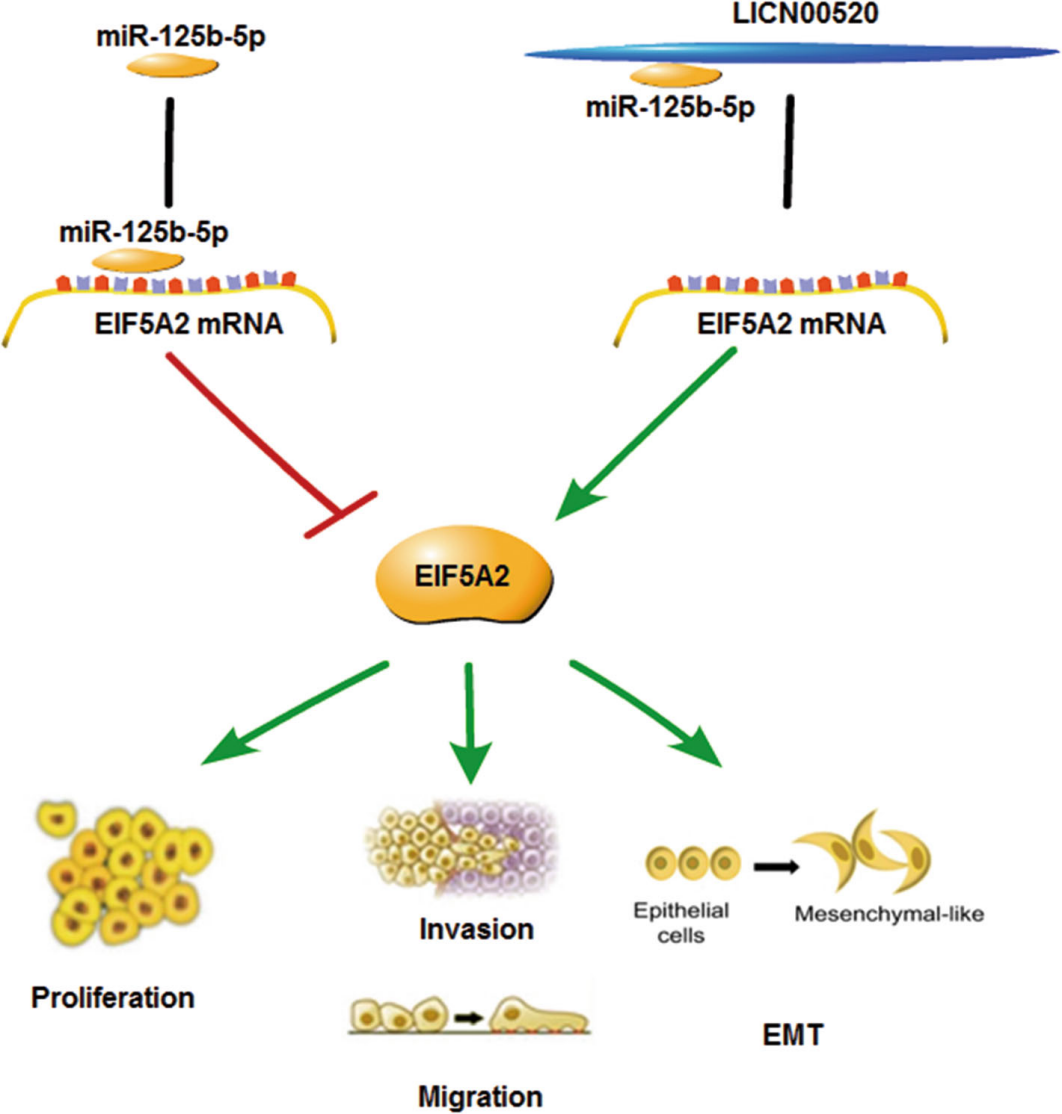
A schematic model showing that LINC00520 facilitates the growth and metastasis of malignant melanoma by competitively binding to miR-125b-5p to liberate EIF5A2 mRNA transcripts

Finally, we studied the effect of LINC00520 on the growth and metastasis of melanoma cells in vivo. Stably expressing LINC00520 shRNA or lentiviral control A375 cells were subcutaneously injected into 10 nude mice (Fig. 8a). After 28 days, the nude mice were sacrificed and the excision tumour is shown in Fig. 8b. Knockdown group of LINC00520 displayed the inhibition of tumor growth compared to the control group between 16 and 28 days (Fig. 8c). The weight of tumour in the LINC00520 shRNA group was lighter than the control group (Fig. 8d). The xenograft tumour tissues were confirmed by H&E staining (Fig. 8e), and the expression of LINC00520, miR-125b-5p and EIF5A2 in the sections of excision tumour were detected. The miR-125b-5p level was increased and the EIF5A2 level was decreased with the knockdown of LINC00520 (Fig. 8f and g). To further investigated the effects of LINC00520 on the metastasis of melanoma cells in vivo, A375 cells that stably expressing LINC00520 shRNA or control were tail vein-injected into nude mice (Fig. 8a). Knockdown group of LINC00520 showed lower levels of lung colonisation compared with the control group (Fig. 8h). Silencing of LINC00520 decreased the number of metastatic lung nodules (Fig. 8i), and HE staining confirmed the metastatic lung tumor tissues (Fig. 8j). On the sections of metastatic pulmonary nodules, miR-125b-5p was increased and EIF5A2 was reduced in the LINC00520 knockdown group (Fig. 8k and l). These results demonstrated that LINC00520 promotes the growth and metastasis of melanoma through regulating miR-125b-5p/EIF5A2 axis (Fig. 9).

## Discussion

Recently, numerous studies have revealed that some lncRNAs play crucial role in the development and progression of many human tumors [24, 31, 32]. LINC00520, located on chromosome 14, is a novel identified lncRNA. LINC00520 has been shown to up-regulate and modulate the malignant phenotype of tumor cells in some malignant tumors [12, 14, 33]. LINC00520 promotes the proliferation, migration and invasion of glioma cells, but inhibits its apoptosis [34]. Wu et al. reported that LINC00520 contribute to the metastasis of laryngeal squamous cell carcinoma [13]. However, the role of LINC00520 in malignant melanoma has not been studied until now. In this study, we first analyzed the lncRNAs expression profile of melanoma tissue, and found that LINC00520 was increased in melanoma. We verified the results of lncRNAs expression profile using more samples, and found that high LINC00520 level conferred a poorer prognosis to melanoma patients based on the analysis of our samples and public database. LINC00520 has also been demonstrated to promote the proliferation, invasion and migration of melanoma cell.

We further explored the mechanism of LINC00520 in melanoma. A handle of studies have been proved that some lncRNAs can act as ceRNAs in the malignant progression of many tumors [35, 36]. ceRNAs reduce the binding of miRNAs to target genes by decoying miRNAs, thus regulating the expression of specific genes [16, 37]. It is reported that LINC00520 exhibits pro-oncogenic function in nasopharyngeal carcinoma by regulating the miR-26b-3p/USP39 axis [14]. We subsequently constructed the LINC00520-miRNA-target gene network based on our miRNA-seq and RNA-seq data and bioinformatics predictions. LINC00520, miR-125b-5p and EIF5A2 were found to have a potential ceRNA correlation in melanoma. Our melanoma samples and public database further confirmed the network of LINC00520, miR-125b-5p and EIF5A2. We demonstrated that LINC00520 directly binds to miR-125b-5p by using Luciferase reporter assay, MS2-RIP assay and RNA pull-down assay. EIF5A2 has also been proved to be the target gene of miR-125b-5p in melanoma. LINC00520 siRNA reperssed the expression of EIF5A2 and the luciferase activity of wild type EIF5A2 3’UTR luciferase vectors, and this repression were attenuated by miR-125b-5p inhibitor. All results suggested that LINC00520 promotes EIF5A2 expression by decoying miR-125b-5p in melanoma.

It has been proved that miR-125b-5p acts as a tumor suppressor in the malignant progress of many human tumors [38, 39]. In particular, miR-125b-5p is an independent predictor of survival in melanoma patients, and miR-125b-5p is down-regulated and suppresses the proliferation and invasion of melanoma [40, 41]. miR-125b-5p were shown to be involved in the vemurafenib resistance of resistant BRAF-mutant melanoma cell [42]. EIF5A2, the member of the EIF family, is a novel oncogene and up-regulated in ovarian cancer, esophageal cancer, gastric cancer, etc. [22, 43–45]. EIF5A2 participates in many biological processes of tumor cells, including growth, metastasis and EMT [19, 22, 45]. It was found that EIF5A2 also plays a role of oncogene in melanoma [23]. Here, we confirmed that miR-125b-5p exert its anti-proliferation and anti-metastasis effects by targeting EIF5A2 in melanoma. Moreover, we demonstrated that the effect of LINC00520 siRNA on the proliferation, EMT, invasion and migration of melanoma cells were reversed by miR-125b-5p inhibitor. LINC00520 also promotes melanoma growth and metastasis in vivo by regulating miR-125b-5p/EIF5A2 axis. We also domnostrated that the role of LINC00520 in melanoma cells is independent of BRAF mutation. Take together, our research reveal the influence of LINC00520/miR-125b-5p/EIF5A2 on the biological progression of melanoma. Huber et al. have reported that miR-125b-5p is released in the circulation and associated with immunotherapy of melanoma [46]. Therefore, to detect the expression level of LINC00520 in blood circulation and explore its value in the clinical diagnosis and treatment of patients with melanoma is our future research direction.

## Conclusion

In conclusion, all results indicated that LINC00520 plays the pivotal role in the development of melanoma. LINC00520 facilitates the growth and metastasis of malignant melanoma by competitively binding to miR-125b-5p to liberate EIF5A2 mRNA transcripts, thereby promotes the EIF5A2 expression. Understanding the molecular mechanism of LINC00520 in melanoma is important to improve our knowledge of the molecular biological of malignant progression of melanoma. The deep study of LINC00520/miR-125b-5p/EIF5A2 axis is helpful for us to identify new biomarkers or therapeutic target for melanoma patients.

## Supplementary information

**Additional file 1: Figure S1.** (A) Western blots identified EIF5A2 protein expression changes in NC, si-LINC00520 or si-LINC00520 plus miR-125b-5p inhibitor transfected MeWo cells, GAPDH was used as a control. (B) Effect of si-LINC00520 on the proliferative ability of MeWo cells was determined by CCK8 assay, and the results were further confirmed by co-transfection miR-125b-5p inhibitor. (C) The invasive capacity of MeWo cells was detected by transwell assay following transfection with NC, si-LINC00520 or si-LINC00520 plus miR-125b-5p inhibitor. (D) The migratory ability of MeWo cells was assessed by the scratch wound assay. miR-125b-5p inhibitor reversed the effect of si-LINC00520 on the migration capability of MeWo cells. Scale bar, 100 μm. Data were expressed as the mean ± SD, *P < 0.05, **P < 0.01, ***P < 0.001.

## Data Availability

All the data and materials supporting the conclusions were included in the main paper.

## Abbreviations

LINC00520: Long intergenic non-protein coding RNA520
EIF5A2: Eukaryotic initiation factor 5A2
LncRNAs: Long non-coding RNAs
CCK-8: Cell counting kit-8
EMT: Epithelial-to-mesenchymal transition
TCGA: The Cancer Genome Atlas
DMEM: Dulbecco’s modified Eagle’s medium
ATCC: American Type Culture Collection
shRNA: short hairpin RNA
siRNA: Small interfering RNA
PI: Propidium iodide
RISC: RNA-induced silencing complex
FISH: Fluorescence in situ hybridization
