# Supplementary figures and images for: Long non-coding RNA LINC00520 promotes the proliferation and metastasis of malignant melanoma by inducing the miR-125b-5p/EIF5A2 axis
Source: J Exp Clin Cancer Res. 2020 May 28;39:96. doi: 10.1186/s13046-020-01599-7 (PMC7254730; doi:10.1186/s13046-020-01599-7)

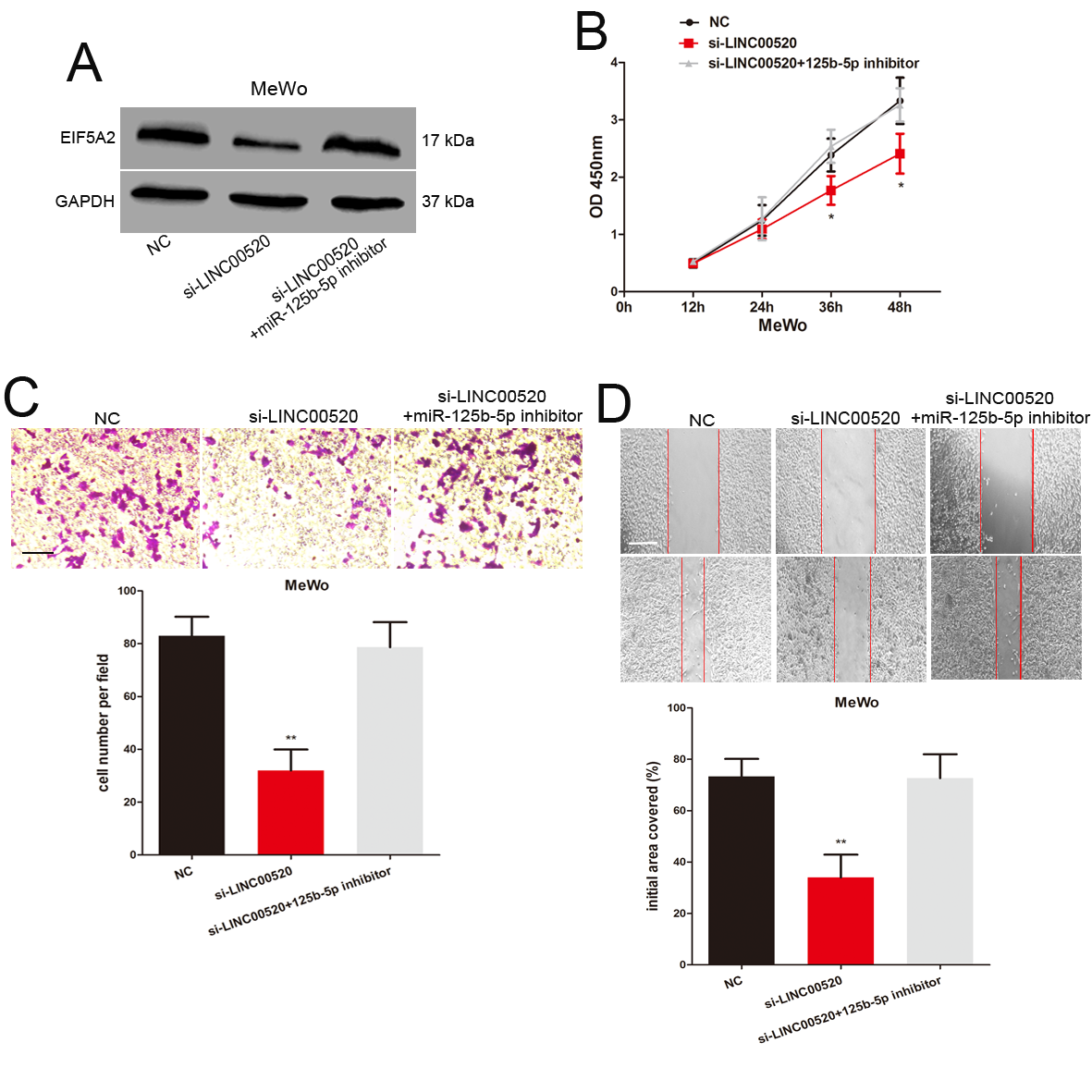

Supplement: Supplementary file 1 — Additional file 1: Figure S1. (A) Western blots identified EIF5A2 protein expression changes in NC, si-LINC00520 or si-LINC00520 plus miR-125b-5p inhibitor transfected MeWo cells, GAPDH was used as a control. (B) Effect of si-LINC00520 on the proliferative ability of MeWo cells was determined by CCK8 assay, and the results were further confirmed by co-transfection miR-125b-5p inhibitor. (C) The invasive capacity of MeWo cells was detected by transwell assay following transfection with NC, si-LINC00520 or si-LINC00520 plus miR-125b-5p inhibitor. (D) The migratory ability of MeWo cells was assessed by the scratch wound assay. miR-125b-5p inhibitor reversed the effect of si-LINC00520 on the migration capability of MeWo cells. Scale bar, 100 μm. Data were expressed as the mean ± SD, *P < 0.05, **P < 0.01, ***P < 0.001. [file 13046_2020_1599_MOESM1_ESM.tif]
